# Beyond the Channels: Adhesion Functions of Aquaporin 0 and Connexin 50 in Lens Development

**DOI:** 10.3389/fcell.2022.866980

**Published:** 2022-04-07

**Authors:** Zhen Li, Yumeng Quan, Sumin Gu, Jean X. Jiang

**Affiliations:** ^1^ Department of Biochemistry and Structural Biology, University of Texas Health Science Center, San Antonio, TX, United States; ^2^ State Key Laboratory of Ophthalmology, Zhongshan Ophthalmic Center, Sun Yat-sen University, Guangzhou, China

**Keywords:** connexin, aquaporin, cell adhesion, lens transparency, lens development, lens cell differentiation

## Abstract

Lens, an avascular tissue involved in light transmission, generates an internal microcirculatory system to promote ion and fluid circulation, thus providing nutrients to internal lens cells and excreting the waste. This unique system makes up for the lack of vasculature and distinctively maintains lens homeostasis and lens fiber cell survival through channels of connexins and other transporters. Aquaporins (AQP) and connexins (Cx) comprise the majority of channels in the lens microcirculation system and are, thus, essential for lens development and transparency. Mutations of AQPs and Cxs result in abnormal channel function and cataract formation. Interestingly, in the last decade or so, increasing evidence has emerged suggesting that in addition to their well-established channel functions, AQP0 and Cx50 play pivotal roles through channel-independent actions in lens development and transparency. Specifically, AQP0 and Cx50 have been shown to have a unique cell adhesion function that mediates lens development and transparency. Precise regulation of cell-matrix and cell-cell adhesion is necessary for cell migration, a critical process during lens development. This review will provide recent advances in basic research of cell adhesion mediated by AQP0 and Cx50.

## Introduction

The lens is an avascular organ formed by epithelial cells at the anterior surface, differentiating fiber cells at the lens equator region, and highly differentiated fiber cells at the lens cortex and core, also called the lens nucleus. During development, epithelial cells at the lens equator continuously proliferate, elongate, and differentiate to lens fiber cells. The highly differentiated fiber cells abruptly lose their organelles to generate an organelle-free zone for clear light transmission. During the differentiation, high concentrations of aquaporin (AQP) 0, crystallins, Connexin (Cx) 46, and Cx50 (Jiang et al., 1995; Petrova et al., 2015) are accumulated to fiber cells. Given that the formation of tissues during embryogenesis largely depends on the close interactions between neighboring cells, the unique structure and organization of the lens organ relies on the maintenance of cell-cell contacts throughout the morphogenetic process. Cell adhesion molecules, E-cadherin and N-cadherin, expressed by undifferentiated lens epithelial cells throughout growth, are thought to be essential for the separation of the initial lens vesicle from the head ectoderm during early lens development (Pontoriero et al., 2009). E-cadherin expression is inhibited after fiber cell differentiation has begun, while N-cadherin is expressed and organized along the fiber cell lateral interfaces in coordination with cortical F-actin (Leonard et al., 2011). As newly differentiating fiber cells turn at the lens fulcrum, they begin to elongate, and their apical surfaces move along the apical surfaces of cells in the adjacent lens epithelium, creating a region defined as the epithelial-fiber interface. Cadherins and catenins-composed adherens junctions maintain the interface, apical-basal polarity, and lens morphology (Cain et al., 2008; Pontoriero et al., 2009; Biswas et al., 2016). In lens, cadherin-forming adherens junctions along their cell-cell borders, pin neighboring fiber cells and regulate lens fiber cell elongation and lens morphogenesis (Cheng et al., 2016; Logan et al., 2017). A study using N-cadherin deficient mice indicated that lens channel proteins might compensate for the loss of cell-cell contacts mediated by N-cadherin as localization of AQP0 increased at lateral cell interfaces of fiber cells, and elevated levels of Cx50 were observed in the subpopulation of migration-defective lens fiber cells in the N-cadherin knock out mice (Logan et al., 2017). Recent advances have begun to shed light on the cell adhesion mediated by AQP0 and Cx50. In this article, we will review recent research advances for the unique adhesive roles of AQP0 and Cx50 in lens homeostasis, transparency, and development.

## Aquaporins and Connexins in the Lens

AQPs and Cxs composed of water channels and gap junctions/hemichannels, respectively, in lens provide fundamental support for lens microcirculation and are pivotal for avascular lens homeostasis, including cell synchronization of intracellular voltage and ion concentrations, differentiation, growth, and metabolic coordination (Mathias et al., 2007). In addition to channel functions, AQPs and Cxs have also been shown to be involved in cell growth, differentiation, and adhesion in a channel-independent manner in the lens or other organs (Gu et al., 2003; Hiroaki et al., 2006; Banks et al., 2007; Kameritsch et al., 2012).

### Aquaporins in the Lens

**FIGURE 1:**
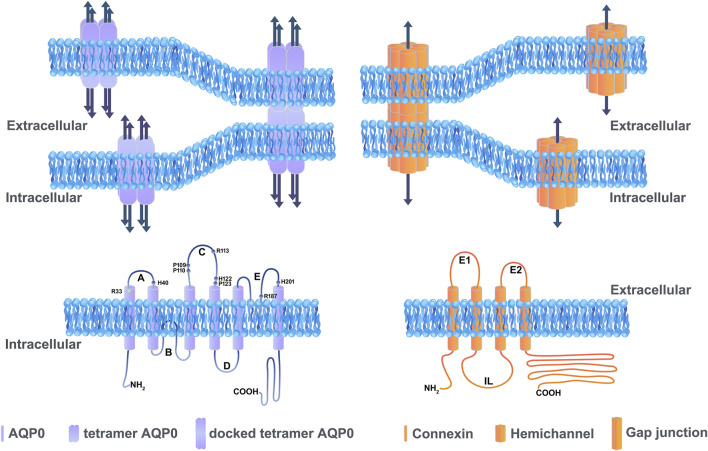
Illustration showing the basic structure of channels formed by aquaporins and connexins. Aquaporins and connexins share similar membrane topologies consisting of transmembrane domains connected by extracellular and cytoplasmic loops; both the amino- and carboxy-termini are intracellular. An AQP0 monomer consists of six transmembrane *α*-helices and two half helices connected by five loops. Each AQP monomer contains an independent water pore. AQP0 tetramers forms wavy thin junctions or square array junctions between adjacent cells. The dark blue arrow represents the pore which transports water (left upper panel). The amino acid residues (Arg33, Arg113, Arg187, His40, His112, His201, Pro109, Pro110, and Pro123) located in the extracellular loop domain involved in the cell adhesion are labeled (left lower panel). The membrane topology of a connexin consists of four transmembrane domains, two extracellular loops and one cytoplasmic loop (right lower panel). Connexin channels provide a pathway for the intracellular and intercellular exchange of ions, small metabolites, and second messengers (dark blue arrow). A hemichannel (also known as connexons) is formed by six connexins that contains a pore in the center, and a hemichannel dock with the other hemichannel of adjacent cells to form a functional intercellular channel termed gap junction channel (right upper panel).

AQPs belong to a small integral membrane protein family with 13 members broadly expressed in various animal cell types (Bill and Hedfalk, 2021). AQP has six transmembrane domains, three extracellular loop domains, two intracellular loop domains with cytosolic N- and C-terminal domains (Figure 1). AQPs form tetrameric membrane-bound channels to facilitate the exchanges of water and some small uncharged solutes, such as glycerol, urea and gas of physiological importance (Gomes et al., 2009; Padhi and Priyakumar, 2020). Among the 13 members of AQPs, three AQP subtypes, AQP0, AQP1, and AQP5, are expressed in the lens. AQP0 is distributed throughout the lens fibers, while AQP1 is located primarily in the lens epithelium (Hamann et al., 1998). AQP5 is expressed in both lens fiber and epithelial cells, decreasing and translocating from intracellular localization to the plasma membrane during lens fiber differentiation (Kumari et al., 2012; Grey et al., 2013). As for AQP8, identified in lens epithelial from cataract patients (Hayashi et al., 2017) will not be discussed here as the mRNA transcript of it was not found in normal eyes (Tran et al., 2013).

AQP0, also known as major intrinsic protein (MIP) 26 in earlier publications (Broekhuyse et al., 1976), was the first sequenced protein among the AQP family and is the most abundant membrane protein in the lens (Hall et al., 2019). But unlike other AQP family members, AQP0 exhibits lower water permeability, at least 40 times lower than that of AQP1 (Chandy et al., 1997). In 1994, MIP26 was identified as a family member of aquaporin through the sequence homology comparison (Verbavatz et al., 1994). The water transport function was confirmed by expressing bovine MIP26 cRNA in *Xenopus* oocytes (Mulders et al., 1995). Structure studies of AQP0 revealed an unusually narrow water-conducting channel with the residue Tyr24 poking into the channel, breaking the hydrogen-bonding pattern of the single-filed water molecules moving through the channel (Roche and Törnroth-Horsefield, 2017). This unique structural feature might partially explain the low water permeability of AQP0. Unlike AQP1 and AQP5, the water permeability of AQP0 is insensitive to mercury compounds (Mulders et al., 1995), but is sensitive to pH and calcium concentration ([Ca^2+^]), with a–4-fold increase in AQP0 water permeability with lower pH or [Ca^2+^] (Németh-Cahalan and Hall, 2000). The calcium regulation of AQP0 is mediated by an interaction with the calcium-binding messenger protein, calmodulin (CaM) (Reichow et al., 2013). The CaM interacts with AQP0 through electrostatic interactions between CaM and the cytoplasmic face of AQP0 or directly binds to a cytosolic arginine-rich loop located near the center of the tetramer of AQP0 (Fields et al., 2017). Thus, element altering the CaM-bonding with AQP0 would result in different calcium regulation of AQP0 water permeability. Since AQP0 accounts for approximately 50% of total membrane protein content (Alcalá et al., 1975), this protein was thought to be important for lens homeostasis and transparency. Indeed, AQP0 gene mutations are directly linked to human congenital cataracts (Sun et al., 2021). AQP0 deletion in a knockout mouse model leads to cataract and spherical aberration phenotypes at 3 weeks of age (Shiels et al., 2001; Kumari et al., 2017). Interestingly, AQP1 is persistently expressed in lens fiber cells of AQP0 knockout mice, which indicates that AQP0 may have unique roles that cannot be adequately fulfilled by other AQPs (Kumari et al., 2017). Given its abundant presence and low water permeability, AQP0 is likely to have water-channel independent functions in the lens.

### Connexins in the Lens

Cx, an integral membrane protein with 21 family members in humans, has four conserved transmembrane domains, two extracellular loop domains, a variable intracellular loop domain, and cytoplasmic N- and C-terminal domains (Beyer and Berthoud, 2018). Six connexins oligomerize to form a connexon (also called hemichannel), and two connexons from two adjacent cells dock with each other to form an intercellular gap junction channel (Figure 1). Many gap junction channels are clustered together to form gap junction plaques. In lens, three Cxs have been identified: Cx43 expressed in lens epithelial cells, Cx46 primarily localized in lens fiber cells, and Cx50 distributed in both lens epithelial and fiber cells (Berthoud and Ngezahayo, 2017). The transcript of a fourth isoform, Cx23, has been detected in the zebrafish embryo and mouse lens (Gustincich et al., 2003; Iovine et al., 2008; Bassnett et al., 2009).

Connexons can consist of either the identical Cx isoforms or a combination of different Cx isoforms, forming homomeric or heteromeric connexons, respectively, (Jiang and Goodenough, 1996). Two adjacent cells may contribute identical or different types of connexons, to form homotypic or heterotypic channels, respectively. Channels formed by different connexins have unique electrical conductance. Heterotypic and heteromeric channels formed by Cx46 and Cx50 display a range of unitary conductance, but none of them ever exceed the homotypic Cx50 value of 220 pS (Hopperstad et al., 2000). *In vitro* studies have unveiled that different conductance of gap junction channels results in different permeability and the intercellular exchange of ions (Na^+^, K^+^, Ca^2+^, and Cl^−^), second messengers [cAMP, cGMP, inositol trisphosphate (IP3)], and small metabolites (glucose, amino acids) (X. Jiang, 2010). In addition, *in vitro* study results suggest that most congenital cataract-related lens Cx50 and Cx46 mutants form non-functional gap junction channels that may impair lens homeostasis through disrupted lens microcirculation (Berthoud et al., 2020). Mutations of Cx46 and Cx50 also induce abnormal hemichannel activities with aberrant voltage-dependent gating or modulation, which can potentially affect the lens microcirculation by depolarizing the cells and decreasing the driving force for the movement of ions throughout the organ (Verselis and Srinivas, 2008; Minogue et al., 2009). Cx46 and Cx50 knockouts result in cataract formation in mice (Shi et al., 2022). In addition, mice with Cx50 deletion exhibits microphthalmia (Rong et al., 2002). Furthermore, studies have showed that knocking-in Cx46 gene in Cx50 gene-deficient mice rescued lens transparency and recovered gap junction coupling and resting voltages with partially improved coupling conductance in differentiating lens fibers, and slightly enhanced postnatal epithelial cell proliferation rates, but microphthalmia remains (White, 2002; White et al., 2007; Wang et al., 2017). There are not obvious phenotypes in Cx43 mutations and Cx43 knockout mouse model. It has been proposed that Cx23 might be expressed in the lens and related to fiber cell differentiation because Cx23-deficient mouse exhibits smaller lenses with opalescent puncta in the nuclear region and a missense mutation of Cx23 (R32Q) in mice results in small eyes, small lenses and polar lens opacities (Gustincich et al., 2003; Iovine et al., 2008). However, Cx23 transcripts and proteins have not been identified in human lenses (Jiang and Goodenough, 1996; Bassnett et al., 2009). Even in the mouse, the cellular distribution of the Cx23 protein is unknown due to the lack of specific antibodies for Cx23. Therefore, we do not include any further discussion of Cx23.

### Aquaporins and Connexins in Lens Development

Lens development is a critical process for eye organogenesis, and its abnormal development results in cataract formation and microphthalmia. During the early stages of lens development, with the thickening of the surface ectoderm overlying the optic vesicle, the epithelial cells in the predetermined region first differentiate to form the lens placode. Invagination of the placode leads to the formation of the lens pit and then the lens vesicle (Piatigorsky, 1981; Li et al., 1994). The cells in the anterior of the lens vesicle become the lens epithelium, and the cells in the posterior of the vesicle form the primary lens fibers through cell elongation and differentiation toward the anterior epithelium. Primary lens fiber cells gradually lose their organelles and rapidly elongate toward the anterior surface to form primary lens fibers. During lens development, secondary lens fibers are subsequently produced by the equatorial epithelial cells that proliferate, elongate and migrate toward the anterior and posterior poles as newly formed fiber cells (Leong et al., 2000). Fiber cells form concentric shells with newly formed fibers in the periphery and mature lens fibers move toward the center of the tissue. The processes of epithelial proliferation and fiber differentiation to form secondary lens fibers lasts throughout the organism’s lifespan (Petrova et al., 2015).

The characteristic shape of the lens depends on highly regulated cell movements during development. The precise regulations of cell-cell and cell-matrix adhesions, and the connections between cell adherence molecules and the cytoskeleton are essential for lens morphogenesis (Zelenka, 2004). Cadherin mediated cell–cell adhesions play a critical role in lens development. During fiber differentiation, cadherins are colocalized with filamentous actin and become increasingly associated with the lens cytoskeleton (Leong et al., 2000). In the process of lens development and growth, the expression patterns of AQPs and Cxs are closely involved in the morphologic differentiation of lens cells. AQP0 is first detected in the membrane of lens epithelium and elongating primary fibers during embryonic lens development at embryonic day (E) 11 (Petrova et al., 2015). AQP0, unlike other members of AQP family, is proposed to serve as a major structural protein to facilitate the formation of the ordered cellular structure (Chepelinsky, 2009). In contrast, during postnatal development, AQP1 serves mainly as a water channel to maintain lens homeostasis (Schey et al., 2017). AQP1 protein expression is detected at E17.5 in lens anterior epithelial cells at a low level and increases at postnatal day 6.5 when the lens microcirculation system is initiated at the later stages of embryonic development (Varadaraj et al., 2007). In addition, AQP5 appears at E10, but is only present inside the cell (Varadaraj et al., 2007; Petrova et al., 2015), which indicates that it may not contribute to water permeability on the plasma membrane at this early stage of embryonic development.

Cx43 is first detected at the lens placode stage (Ookawara et al., 1992), while Cx46 and Cx50 are first synthesized at the vesicle stage along with the primary fiber elongation (Evans et al., 1993; Jiang et al., 1995). Cxs undergo posttranslational modifications, such as protein phosphorylation in the cortical fibers (Saleh et al., 2001; Wang and Schey, 2009) and the increased proteolytic cleavage of the COOH termini from cortical fibers to outer cortex to nucleus (Slavi et al., 2016) which resulted in and reorganization with gap junctional plaque (Biswas et al., 2009) and function in mature nuclear fibers (Sheng Lin et al., 1998; DeRosa et al., 2006; Liu et al., 2011a). In addition to their roles in lens hemostasis and microcirculation, unlike Cx46, Cx50 plays a critical role in lens fiber differentiation and lens development. Cx50 gene knockout mice exhibit microphthalmia in addition to zonular pulverulent cataracts (White et al., 1998). An earlier study from our lab shows that overexpression of Cx50 promotes lens fiber cell differentiation in primary chick lens culture (Gu et al., 2003). Moreover, we found that Cx50 retains Skp2, an E3 ligase in the cytosol, which will prevent Skp2 from migrating into the nucleus and degrades cell cycle inhibitors, p57/p27, leading to cell cycle arrest and ultimately inhibiting cell proliferation and promoting fiber cell differentiation (Shi et al., 2015).

## Mechanism of AQP0 Adhesive Function

### Homotypic Adhesion

As mentioned above, besides its channel functions, AQP0 also plays a unique and crucial role as an adhesion molecule in mediating the formation of thin junctions between lens fibers (Figure 1). Thin section transmission electron microscopy (TEM) analyses of AQP0 show that in mature fiber cells, AQP0 forms wavy thin (10 nm) junctions or square array junctions between adjacent fiber cells, indicating AQP0 may possess cell adhesive properties due to the interaction between AQP0 molecules from opposing plasma membranes (Lo and Harding, 1984). Consistent with the observation by TEM, double-layered 2D crystals obtained from the reconstitution of AQP0 isolated from the core of sheep lenses display the same dimensions as the thin 11-nm lens fiber cell junctions (Gonen et al., 2004). AQP0 membrane junction formed by localized interactions between AQP0 tetramers is mainly mediated by proline residues in the extracellular loop domains A and C (Figure 1). These proline residues in the extracellular loop domain A and C are evolutionarily conserved in AQP0 but not conserved in most of the other aquaporin isoforms. It is also shown that Pro109, Pro110, Arg113, and Pro123 in the C-loop domain are involved in the interaction between two AQP0 molecules on membranes of adjoining cells (Engel et al., 2008). In addition, AQP0 can be pulled down with the glutathione S-transferase (GST)-AQP0-C-loop and GST-AQP0-C terminals, but not with the GST-AQP0-A-loop, GST-AQP0-E-loop, or GST alone (Yu et al., 2005). This protein pull-down study confirms that the intercellular adhesion by opposing AQP0 occurs in the extracellular loop domains. Moreover, the intracellular C-terminal domain appears to mediate the formation of AQP0 tetrameric oligomers within the cell (Nakazawa et al., 2017). *In vitro* cell adhesion assay using mouse fibroblasts lacking endogenous adherence molecules unveils the cell adhesive role of AQP0 (Kumari and Varadaraj, 2009). Contrary to mammalian AQP0, zebrafish have two AQP0 isoforms, AQP0a and AQP0b, and AQP0b has strong adhesive properties while AQP0a does not (Vorontsova et al., 2021). Consistent with biochemical data, mutation of zebrafish AQP0b N110T (N110 is equivalent to P110 in mammalian AQP0) decreases the percentage of adherent cells due to homotypic adhesion of AQP0b (Michea et al., 1994). The mutation T110N in AQP0a, which makes the identical residue as AQP0b at the same location, increases adhesive properties similar to WT AQP0b. These studies highlight the importance of residue Pro110 in AQP0 cell-to-cell adhesion (Vorontsova et al., 2021). Furthermore, swapping loop C of AQP0 with that of AQP1 resulted in plasma membrane localization of AQP0. Together, the loop C domain of AQP0 is identified as a critical domain for the homotypic adhesion function of AQP0.

### Heterotypic Adhesion

In addition to AQP0-AQP0 homotypic interaction, a AQP0-plasma membrane interaction has been also proposed. A biophysical study with AQP0-containing proteoliposomes shows a resonance energy transfer and an increase in turbidity occurred only with the presence of both AQP0 and phosphatidylserine vesicles. This study indicates the heterotypic adhesion between AQP0 and a negatively charged membrane (Michea et al., 1994). It is also suggested that the AQP0-plasma membrane interaction is electrostatic in nature, meaning the requirement of several positively charged residues in the AQP0 extracellular loops A, C, and E. The positively charged protein surface of mouse AQP0 is made of three arginine residues (Arg33 in loop A, Arg113 in loop C, and Arg187 in loop E) and three histidine residues (His40 in loop A, His122 in loop C, and His201 in loop E) in extracellular loop of AQP0. Among these arginine residues, a mutation at Arg33, R33C was also identified as a mutation causing autosomal dominant congenital lens cataracts in a five-generation Chinese family (Gu et al., 2007)and shows reduced cell–cell adhesion, but normal protein localization and water permeability (Kumari et al., 2013). In addition, another study reported that the adhesion function decreased in the mutations of all positively charged residues in loops A and C of AQP0 (Kumari et al., 2019). It is worth noting that the mutation of Arg113 to Gln decreases the AQP0 adhesion by changing the positively charged residue to neutral (Kumari et al., 2019). However, in another study, the mutation of Arg113 to Gly, another neutral amino acid, did not affect cell-cell adhesion (Nakazawa et al., 2017). The data generated from a double-layered 2D crystal structural study provides a possible explanation; the Arg113 in loop C from AQP0 monomer in one cell interacts with Pro123 in loop C from the AQP0 monomer in the adjacent cell to facilitate AQP0-AQP0 interaction (Engel et al., 2008). The changes in hydrophobicity or hydrogen bond in the Arg 113 to Gly mutation might compensate for the less adhesion introduced by electrostatic interaction. These studies suggest that substitution of uncharged residues for positively charged residues inhibits AQP0-mediated cell adhesion, which offers solid support concerning the interaction between the positively charged residues of AQP0 and negatively charged opposing plasma membrane through electrostatic interaction.

Evidence of the role the C-terminal domain of AQP0 plays in cell adhesion has been contradictory. Researchers first proposed that the C-terminal truncated AQP0 was the adhesive form while full-length AQP0 primarily fulfills the role of a water channel (Engel et al., 2008). This hypothesis is based on the fact that full-length AQP0 yields more single-layered 2D AQP0 crystals while C-terminal truncation primarily induces double-layered 2D AQP0 crystals (Gonen et al., 2005). High-resolution structure analysis supports this hypothesis. The X-ray structure at the resolution of 2.2 Å reveals that the tetramer formed by C-terminal truncated AQP0 has a reconfigured loop A domain which positions Pro38 towards a rosette-like structure at the center of the tetramer and facilitates a major junctional contact. C-terminal truncation also causes a swapped position in the side chains of Arg33 with Trp34, leading to the proximity of another tetramer compared with intact AQP0 (Gonen et al., 2005). However, the role of C-terminal truncated AQP0 in cell adhesion was not supported by experimental evidence using adhesion function studies. Mouse fibroblast cells expressing full-length AQP0 exhibit cell-cell adhesive function (Kumari and Varadaraj, 2009). A protein pull-down study provides direct evidence that the C-terminal domain is important for the interaction between AQP0’s (Nakazawa et al., 2017). Based on the above evidence, C-terminal truncation would be expected to compromise the adhesive function. Intriguingly, fibroblast cells expressing C-terminal truncated AQP0 (1–243, 1–246, 1–249, and 1–259) exhibit comparable levels of adhesion compared to full-length AQP0 (Sindhu Kumari and Varadaraj, 2014). AQP0 1–243 truncation appears to be less adhesive, but after normalization with membrane protein expression, there is no difference between AQP0 1–243 and full-length AQP0. However, the length of C-terminus required for cell adhesion could not be determined since further truncation mutant proteins like AQP0 1–234 and AQP0 1–238 failed to localize on the plasma membrane. It is difficult to firmly establish that the C-terminal domain directly participates in AQP0-plasma interaction. Further studies also show that there is no difference between AQP0 1–246 and full-length AQP0 either in homotypic or heterotypic pairing in fibroblasts (Varadaraj and Kumari, 2018). Fibroblast cells expressing full-length AQP0 or AQP0 1–246 adhere to negatively charged l-α-phosphatidylserine lipid vesicles, but not to neutral phosphatidylcholine lipid vesicles. However, works from the same group demonstrate that lens fiber cell membrane vesicles prepared from the mice expressing AQP1-246 show an increased adhesion to mouse fibroblast cells compared with WT AQP0 (Kumari and Varadaraj, 2019).

Interestingly, AQP0 1–246 in different model systems mentioned above exhibit variable adhesive properties, which might be caused by the influence of the lipid microenvironment on AQP0 protein. First, the high-resolution density maps show that reconstituted AQP0 with the anionic lipid dimyristoyl phosphatidylglycerol yields a mixture of 2D crystals with different symmetries, while reconstitution of AQP0 with dimyristoyl phosphatidylserine yielded a crystal with typical symmetry (Hite et al., 2015). Reconstruction of AQP0 with different lipids exhibit different levels of symmetries, indicating lipids might create distinct membrane surface properties that could modify the properties of the embedded membrane proteins. Second, the freeze-fracture structure of AQP0 in lipid vesicles shows individual intramembrane particles at a low protein/lipid molar ratio (1:20,000); some protein clustering at a higher protein/lipid ratio (1:400), and large aggregates or two-dimensional crystalline regions of AQP-0 at an even higher protein/lipid ratio (1:100) (Ehring et al., 1990). Detergent extraction analysis and confocal microscopy imaging show similar results that AQP0 is located almost exclusively in the detergent soluble membrane at a 1:1200 AQP0/lipid ratio, whereas half of the AQP0 protein is sequestered into detergent-resistant membranes and oligomerizes at a 1:100 ratio (Tong et al., 2009). These results infer that different lipid environments could significantly impact AQP0 localization in the plasma membrane. Lastly, molecular dynamics experiment indicated that AQP0 protein surfaces induce specific fluid- and gel-phase prone areas at room-temperature, and several lipid layers might guide AQP0 interactions towards other membrane components (Briones et al., 2017). This model is compatible with the squared array oligomerization of AQP0 tetramers separated by a layer of annular lipids (Briones et al., 2017). Another interesting phenomenon is that there is no difference between lens fiber cell membrane vesicles prepared from lens outer cortex or inner cortex in their adhesion to fibroblast cells (Varadaraj and Kumari, 2018). This observation supports the notion that full-length and C-terminal truncated AQP0 might have comparable adhesive function since C-terminal truncation is primarily detected in the core of the lens while integral AQP0 was more expressed in the outer cortex (Grey et al., 2009; Wenke et al., 2015). However, there are several possible caveats: First, the age of mice used in this study is not specified. At postnatal day 21, C-terminal truncation was detected in lens core fiber but not lens cortex in mice (Grey et al., 2009). Second, cell adhesion properties possessed by other proteins in lens fiber cells, such as Cx50 cannot be excluded, which will be discussed separately in the later sections. Together, although the underlying molecular mechanism of homotypic adhesion by AQP0 remains largely elusive, heterotypic adhesion is likely mediated by the positively charged loop C domain of AQP0, which interacts with the negatively charged molecules of opposing plasma membranes through electrostatic interactions to form thin adherens junctions in the lens.

## AQP0 Adhesion in Lens Structure and Transparency

Studies in transgenic mouse model revealed that the stiffness of the lens correlates with the expression level of AQP0 protein, indicating that AQP0 is likely required for maintaining optical quality and biomechanical properties of the normal lens (Sindhu Kumari et al., 2015). Lenses expressing only 50% of AQP0 protein have significantly lower optical refracting power than wild-type (WT) lenses, and these lenses scatter light and exhibit spherical aberration (Kumari and Varadaraj, 2014). Loss of AQP0 in mice induced not only worse optimal focusing compared with 50% loss of it but also cataract formation (Shiels et al., 2001). Cataract formation could be observed at embryo day 17.5 is related with fiber cell degeneration. These defects reflect the essential roles of AQP0 in lens fiber cell differentiation and lens transparency. Earlier studies showed low water permeability in the opaque lens of mice expressing null AQP0 (Shiels et al., 2001) and a chimeric AQP0, comprised of AQP0 with an ETn long terminal repeat sequence (Shiels et al., 2000; Kalman et al., 2006), highlighting the importance of the water channel function of AQP0 for lens transparency. However, compromised adhesion was also observed in AQP0 knock out mice in the studies (Kumari et al., 2011). Thus which function of AQP0 is responsible for lens transperancy? The adhesion function, but not water permeability, appears to play a major role in lens structure and transparency. Replacement of AQP0 with AQP1 improved the shape, structure, and outer cortical fiber cell transparency by rescuing and enhancing water channel function, which is 2.6 fold higher than the wild-type lenses. However, the replacement of AQP0 with AQP1 was still ineffective in restoring lens transparency, the normal fiber cell interdigitations and Y-suture formation (Varadaraj et al., 2010). In the meantime, mice with AQP1 overexpression in fiber cells do not exhibit any difference in size, transparency, or light-focusing ability compared with WT mice, indicating that lens could maintain its development and clarity with the higher water permeability of AQP1 in the lens core and cortex (Kumari et al., 2011). Aqp0-R33C, a congenital cataract mutation of AQP0, showed proper intracellular trafficking, membrane localization and water permeability like WT-AQP0 (Kumari et al., 2013). As discussed above, change of positively charged residue in AQP0 extracellular surface induced less AQP0-membrane interaction likely by interrupt electrostatic interaction. The Arg33 is one of the positively charged residues on the surface. This result suggests that adhesion defect is a result of interrupted electrostatic interaction. Nevertheless, R33C with impeded adhesion function only results in disorganized lens fiber cells and cataract formation (Kumari et al., 2013). It is less clear whether the adhesion defect is downstream of the cell-cell communication dysfunction. Therefore, the adhesion function of AQP0 is necessary for regaining complete lens clarity. In addition to transgenic mice, gene knockdown experiments in zebrafish also confirmed the importance of cell adhesion as morpholino knocking down the AQP0b gene, an adhesion subtype of AQP0 found in zebrafish, results in cataract formation (Froger et al., 2010). This defect could be rescued by injection of exogenous cDNA of AQP0b, but not AQP0a, an AQP0 subtype that forms functional water channel in zebrafish (Clemens et al., 2013). Subsequently, CRISPR-Cas9 knockouts of Aqp0a and Aqp0b in zebrafish provided detailed mechanisms regarding how loss of AQP0a and AQP0b induced a disrupted refractive index gradient in lens and confirmed the morpholino findings that both proteins are essential for lens development and transparency (Vorontsova et al., 2018). However, heterogeneric AQP0b exhibited worse optical focusing indicating the importance of adhesion in lens transparency (Vorontsova et al., 2018).

AQP0 was also proposed as a specific adhesion molecule required for the normal structure of interlocking protrusions, a major structural feature in maintaining fiber cell stability. Immunogold labeling shows that interlocking protrusions have significantly more AQP0-labeled gold particles than adjacent flat membranes (Lo et al., 2014). Subsequently, scanning electron microscopy shows that the interlocking protrusions in mature fiber cells of Aqp0 knockout mice are elongated, deformed, and fragmented during fiber cell differentiation, and this maturation resulted in fiber cell separation, breakdown, and cataract formation in the lens core region. Transmission electron microscopy demonstrated that transgenic mice overexpressing AQP1 could compensate the loss of membrane water permeability in the AQP0 KO in the lens fiber cells, but could not restore the characteristic architecture and compact packing of lens fibers (Varadaraj and Kumari, 2018).

## Adhesion Function of Cx50 in Lens Development

The cell adhesive function of Cxs has been proposed over the decades due to their formation of gap junctions between adjacent cells and close association with other adhesion-related proteins, including tight junction proteins, cadherins, and other cytoskeletal proteins (Wu and Wang, 2019). For example, gap junctions formed by Cx43 are suggested to function analogously to cell adhesion molecules in mediating cellular recognition, selective neurite adhesion and repulsion by its interaction with the actin cytoskeleton (Strauss and Gourdie, 2020). The unique role of Cx50 in cell adhesion was identified in a study investigating the functional relationship between Cx50 and AQP0 (Hu et al., 2017). The initial goal of our study was to determine if Cx50 had any impact on the cell adhesion function of AQP0. Interestingly, we found that, like AQP0, exogenous expression of Cx50 in fibroblast cells increased adhesive properties, while the other two Cxs expressed in the lens, Cx43 and Cx46, failed to exhibit any increase of adhesion. To control for possible involvement of gap junction formation in cell adhesion function, a cell-to-cell adhesion assay was performed through the heterotypic pairing of Cxs. Interestingly, compared to non-Cx expressing cells, expression of Cx43 and Cx46 does not further increase adhesion function with heterotypic pairing of the cells expressing Cx50. Thus, like AQP0, Cx50 is capable of mediating both homotypic interaction and heterotypic protein-membrane interaction (Hu et al., 2017). Furthermore, a Cx50 mutant, P88S, a site mutation that impairs the ability of Cx50 to form functional gap junction channels and hemichannels (Berry et al., 2020), exhibits comparable cell adhesive capability as WT Cx50 (Hu et al., 2017). Thus, this experimental evidence suggests that Cx50, but not the other lens Cxs, acts as a cell adhesive molecule, and this adhesive function is independent of its role in forming Cx channels. We identified that the extracellular E2 domain of Cx50 is responsible for cell adhesion function (Hu et al., 2017). We showed that the second extracellular domain (E2) of Cx50 is responsible for its adhesion function because blocking this domain using a mimetic peptide and GST fusion protein conjugated E2 domain containing site mutations resulted in decreased adhesion (Hu et al., 2017). Protein pull-down assay confirmed that the Cx50 E2 domain but not E1 domain mediate intermolecular interaction of Cx50 (Hu et al., 2017). Lens fiber differentiation indicated by lentoid numbers and AQP0 expression was reduced in chick lens primary cultures treated with a fusion protein of the Cx50 E2 domain (Hu et al., 2017). However, E2 domain fusion protein containing site mutations that interrupt cell adhesion fails to attenuate the inhibitory effect of E2 domain on lens fiber differentiation. These studies assert that Cx50-mediated cell adhesion plays a critical role in lens epithelial to fiber cell differentiation and lens development.

## Relationship Between AQP0 and Cx50 in Cell Adhesion

Cx50, but not Cx46 or Cx43, was proved to interact with AQP0 during the early stages of embryonic lens development (Yu and Jiang, 2004; Yu et al., 2005). Protein pull down assay revealed that the intracellular loop (IL) domain of Cx50 and the CT domain of AQP0 directly interact with each other (Liu et al., 2011b). IL domain swapping studies of Cxs further confirmed the specific interaction between AQP0 and Cx50 as embryonic chick lens expressing exogenous wild-type Cx50 co-localizes with endogenous AQP0 while Cx50 mutant with IL domain swapped with that of Cx43 does not co-localize with AQP0 (Liu et al., 2011b). In addition, co-expression of Cx50 and AQP0 in mouse fibroblast cells increased 20–30% of the intercellular coupling and electrical conductance of Cx50 gap junctions compared with Cx50, indicating Cx50-AQP0 interaction promotes gap junction function (Liu et al., 2011b). It is postulated that the cell-to-cell adhesion function of AQP0 serves to increase the proximity of adjacent fiber cells, thus enhancing the probability of gap junction formation. In primary chick lens fiber cell culture, exogenous co-expression of AQP0 with Cx50 enhanced gap junction coupling while treatment with the GSP-fusion protein of AQP0 containing extracellular loop domains disrupted adhesive properties and impeded the enhancement on gap junctions (Liu et al., 2011b). Since gap junction coupling is increased between cells, it is expected to show an increase in adhesion of the cells expressing both AQP0 and Cx50 (Hu et al., 2017). Co-expression of Cx50 and AQP0 has greater cell adhesion enhancement than the sole expression of Cx50 or AQP0 (Hu et al., 2017). The adhesion enhancement observed in Cx50 was absent in fibroblast cells co-expressing Cx43 or Cx46 with AQP0. As AQP0 interacts with Cx50 through its C-terminal domain (Yu and Jiang, 2004), the fusion protein of AQP0 without C-terminus appears to have no obvious effect on this interaction as AQP0 without C-terminus was unable to pull down proteins from lens lysate. Gap junction coupling was also reduced in both AQP0-deficient and AQP0 ΔC/ΔC transgenic mice with C-terminally end-cleaved aquaporin 0 expressed in fiber cells (Al-Ghoul et al., 2003; Varadaraj et al., 2019). Lens fiber cell membrane vesicles from AQP0 ΔC/ΔC transgenic mice exhibited comparable water permeability to wild type mice (Kumari and Varadaraj, 2019) indicating that gap junction coupling was regulated by AQP0 C-terminals and its adhesion function.

**FIGURE 2:**
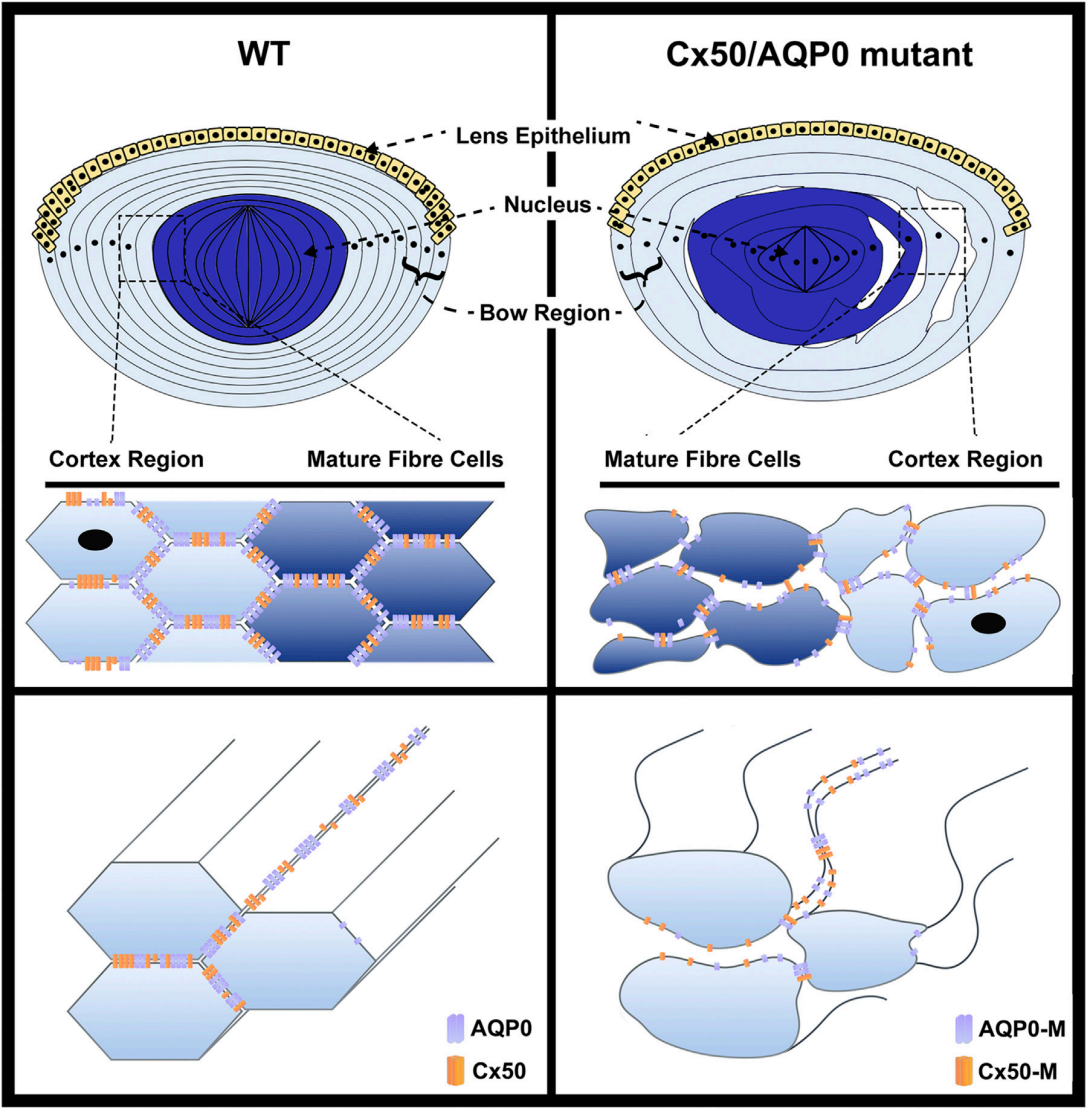
Impairment of cell adhesive function mediated by AQP0 or Cx50 mutation disrupts the organization of lens fibers. In lens fibers, AQP0 and Cx50, primarily located at the short side and broad side of mature lens fibers, respectively, directly interact with each other, and function like “glue” to mediate the cell–cell adhesion, which maintains lens fiber integrity and organization (left upper and lower panels). The hexagon shape indicates cross-section of lens fiber cells. The lenses showing mutations of Cx50 or AQP0 lose cell–cell adhesion in fiber cells, resulting in the impairment of epithelial cell proliferation, increased intercellular spaces and disorganization of lens fibers, which consequently leads to loss of lens elasticity and transparency (right upper and lower panels). The complete loss of Cx50 or AQP0 (Cx50 KO or AQP0 KO) lead to a similar levels of fiber cell disruption.

The interlocking system between fiber cells consists of protrusions, balls, and sockets in the lens of various species studied (Kuwabara, 1975; Kuszak et al., 1980; Willekens and Vrensen, 1982). As aforementioned, AQP0 participates in the formation of protrusions and Cx50 appears to be responsible for regulating surface ball and socket structures based on the following evidence. First, electron microscopy revealed that gap junctions are selectively associated with interlocking balls and sockets in various species (Biswas et al., 2010). Disruption ball and socket structures was observed in Cx50 knockout mice, but not in Cx46 knockout mice (Wang et al., 2016). Lastly, most regions of Cx46 knockin lenses showed little to no of ball and socket structures, indicating Cx46 knockin could not rescue the disruptions caused by Cx50 knockout (Wang et al., 2017). Cell adhesive functions by AQP0 and Cx50 at the short side and broad side of mature lens fiber, respectively, functions like “glue” to maintain lens fiber integrity and organization (Figure 2). Thin-section electron microscopy revealed the presence of numerous intercellular spaces between the lens fiber cells and loss of lens fiber membrane structure in AQP0 or Cx50 gene knockout mice, and this structure disruption appears to be more severe in double gene knockout mouse lenses with the deletion of both AQP0 and Cx50 genes (Gu, et al., 2019).

## Future Prospective and Challenges

Cell culture, chicken lens primary culture, and mouse models with various genetic manipulations have been established, allowing us to obtain invaluable knowledge of the uncanonical roles of AQP0 and Cx50 in cell adhesion in the lens. While significant progress and advances have been made in the last decade, there are still many unanswered questions in our understanding of the adhesion function mediated by AQP0 and Cx50 in lens homeostasis, lens transparency, and development. The detailed molecular mechanism of adhesion of Cx50 and AQP0, especially the heterotypic adhesion between protein and plasma membrane, is yet to be unveiled. Furthermore, one of the future challenges is related to the mechanistic elucidation of the regulatory role of adhesion played by these two proteins during lens fiber cell differentiation and lens development.
